# Monitoring the Response to Tyrosine Kinase Inhibitor (TKI) Treatment in Chronic Myeloid Leukemia (CML)

**DOI:** 10.4084/MJHID.2014.009

**Published:** 2014-01-01

**Authors:** Ibrahim C. Haznedaroglu

**Affiliations:** Hacettepe University Medical School, Department of Hematology, Ankara-Turkey

## Abstract

The aim of oral tyrosine kinase inhibitor (TKI) treatment in chronic myeloid leukemia (CML) is to get ideal hematological, cytogenetic, molecular responses at the critical time points. The depth of the response obtained with TKI and the time to achieve this response are both important in predicting the prognosis in patients with CML. The high efficacy of the TKI treatment of CML has prompted the need for accurate methods to monitor response at levels below the landmark of CCyR. Quantification of BCR-ABL transcripts has proven to be the most sensitive method available and has shown prognostic impact with regard to progression-free survival. European LeukemiaNet (ELN) molecular program harmonized the reporting of results according to the IS (International harmonization of Scale) in Europe. The aim of this review is to outline monitoring the response to optimal TKI treatment based on the ELN CML 2013 recommendations from the clinical point of view as a physician. Careful cytogenetic and molecular monitoring could help to select the most convenient TKI drug and to optimize TKI treatment. Excessive monitoring may have an economic cost, but failure to optimize TKI treatment may result in CML disease acceleration and death.

## Introduction

Current standard therapy for chronic phase (CP-) Ph+ Chronic myeloid leukemia (CML) is the chronic oral administration of tyrosine kinase inhibitor (TKI) drug.^1^ European LeukemiaNet (ELN) 2013 recommendations provided clear, practical suggestions for the physicians dealing with CMLmanagement, based on the best available evidence about the TKI drugs, without disregarding clinical realities and expectations.^1^ The aim of this review is to outline monitoring the response to optimal TKI treatment based on the ELN CML 2013 recommendations from the clinical point of view as a physician.

Based on the true ELN philosophy, the cost of monitoring is much lower than the cost of the TKI drugs.

Careful cytogenetic and molecular monitoring could help selecting the most convenient TKI drug and to optimize TKI treatment.^1^ Excessive monitoring may have an economical cost, but failure to optimize TKI treatment may result in CML disease acceleration and death. Insufficient diagnostic/therapeutic clinical intervention during the management of CML disease course with TKI drugs can cause accelerated phase (AP) or blastic crisis (BC). The survival after the progression into AP/BC is still significantly shorter even in the powerful TKI era.^2^

## Diagnostic Tools and Surrogate Markers for the Monitoring the Response to TKI in CML

Ph+ CML disease burden should be monitored during the TKI treatment.^3^ Hematologic response (HR) is measured by the evaluation of complete blood counts (CBC), white blood cell differential (WBC), and assessment of the spleen size. The definition of the hematologic, cytogenetic and molecular responses are depicted in Table 1. Cytogenetic response (CyR) is detected via the chromosome banding analysis of the bone marrow cell metaphases. The principle of the molecular response (MR) depends upon the measurement of the BCR-ABL transcript levels relative to a control gene. After one year of TKI treatment in CML, complete (C) HR can be obtained in about 98%, CCyR in 57–88%, and major (M)MR in 18–58% of the patients.^1^,^4^–^6^

## Optimal Cytogenetic and Molecular Monitoring in CML Based on ELN 2013 Recommendations

The responses to TKI in CML can be assessed either with molecular tests alone or with cytogenetic tests alone, depending on the local laboratory facilities.^1^,^7^–^14^ However, both cytogenetic and molecular tests are recommended, until a CCyR and an MMR are achieved. Then quantitative molecular tests from the peripheral blood samples alone may be sufficient.^1^

The molecular ELN CML 20131 recommendations are; quantitative RT-PCR of blood cells every 3 months, until the MMR is achieved and confirmed, and then RT-PCR every 3 to 6 months. The molecular results must be expressed by the IS (International harmonization of Scale).^1^

The cytogenetic ELN CML 20131 recommendations are; chromosome banding analysis (CBA) of marrow cell metaphases at 3 and 6 months, then every 6 months until the CCyR is achieved. CBA of the bone marrow cells should be repeated at least every 12 months only if the molecular response cannot be measured. FISH of the blood cells can substitute for CBA only if bone marrow cells cannot be obtained, and only for the definition of CCyR.^1^

Mutational analysis is recommended in case of progression, failure and warning based on the ELN CML 2013^1^,^15^ recommendations. In case of failure, warning, and of development of myelodysplastic features (unexpected leukopenia, thrombocytopenia, or anemia), CBA of the bone marrow cell metaphases is recommended.^1^

## Monitoring TKI Response at the Critical Time Points in CML Based on ELN 2013 Recommendations

At the diagnosis of CML; CBA of the marrow cell metaphases, FISH in case of Ph negativity, to identify variant, cryptic translocations and qualitative PCR (identification of transcript type) are required.^1^

During the treatment of CML; Quantitative, real-time PCR (RQ-PCR) for the determination of BCR/ABL^1^ transcripts level on the international scale, to be performed every 3 months until an MMR has been achieved, then every 3 to 6 months and/or CBA of the bone marrow cell metaphases (at least 20 banded metaphases), to be performed at 3, 6 and 12 months until a CCyR has been achieved, then every 12 months. Once a CCyR is achieved, FISH on blood cells can be used. If an adequate molecular monitoring can be assured, cytogenetics can be spared.^1^

In the case of failure or progression of CML; RQ-PCR, mutational analysis, and CBA of the bone marrow cell metaphases and immunophenotyping in blastic phase are required.

When a ‘Warning’ sign appeared during the TKI administration in CML based on ELN 2013; Molecular and cytogenetic tests to be performed more frequently. CBA of the bone marrow cell metaphases recommended in case of myelodysplasia or complex karyotypic abnormalities (CCA)/Ph+ with chromosome 7 involvement.^1^

## Ideal Response Level to the TKI Treatment Detectable During the Long-Term Monitoring in CML

The ideal responses to the TKI treatment detectable during the long-term monitoring of CML are depicted in Table 2. Inability to detect ELN-warnings in a CML patient receiving a given TKI, resulting in drug failure and/or disease progression can cause damage to the patient.^16^ Proper therapeutic interventions in case of primary and secondary failures during the TKI treatments are described in the ELN 2013 recommendations.^1^

## Clinical Significance of the Ideal Response Level to the TKI Treatment Detectable During the Long-Term Monitoring in CML

The aim of TKI treatment in CML is to get ideal hematological, cytogenetic, molecular responses in the critical time-points (at the 3rd month, at the 6th month, after one year, and thereafter) as depicted in Table 1. The depth of the response obtained with TKI and time to achieve this response are important for the prediction of prognosis in the patient with CML.^16^ Clinical significances of the ideal response level to the TKI treatment detectable during the long-term monitoring in CML are indicated below.

*CHR;* complete hematological response is defined as normal CBC, normal peripheral blood smear and normal spleen in the physical examination.^17^ CHR is the first station during the TKI treatment and must be obtained less than 3 months and should be maintained during the long-term management of CML. CHR can be achieved in about 98% of the patients with CML in the TKI era. Absence of CHR at any stage during the CML disease course is a clear sing of disease progression. Proper therapeutic intervention in the absence of CHR during the TKI treatments is described in the ELN 2013 recommendations.^1^

*CCyR;* complete cytogenetic response is defined as the absence of Ph+ chromosome in the CBA of the bone marrow cells in at least 20 banded metaphases. CCyR is the golden standard during the TKI treatment and must be obtained within the first year (ideally at the six months of TKI regimen) and should be maintained during the long-term management of CML. CCyR is a significant barrier against the CML disease progression. CCyR can be achieved in about 57–88% of the patients with CML in the TKI era. Absence of CCyR after one year of CML disease course is a great danger for disease progression. Proper therapeutic intervention in the absence of CCyR during the TKI treatments is described in the ELN 2013 recommendations.^1^

*MMR;* major molecular response is defined as BCR-ABL ≤ 0.1% in the quantitative RT-PCR of blood cells. MMR is a safe haven during the TKI treatment and must be obtained within the 18 months (ideally at the 12th months of TKI regimen) and should be maintained during the long-term management of CML. MMR is a very significant barrier against the CML disease progression. MMR can be achieved in about 18–58% of the patients with CML in the TKI era. Absence of MMR after 18 months of CML disease course is a danger for disease progression. Proper therapeutic intervention in the absence of MMR during the TKI treatments is described in the ELN 2013 recommendations.^1^

*EMR;* early molecular response is defined as BCR-ABL/ABL ≤ 10% cut-off in the quantitative RT-PCR of blood cells.^18^,^19^ EMR (ideally at the 3^rd^ month of TKI treatment) can predict long-term prognosis during the TKI treatment and must be reached within the first 6 months during the management of CML.^8^,^16^,^20^ EMR is a prognostic sign for CML disease course. EMR can be achieved in about 91% of the patients with CML receiving nilotinib and 67% receiving imatinib in the ENESTnd trial.^21^ Absence of EMR after 6 months of CML disease course represents an aggressive disease course in the long-term for instance after 5 years. Proper therapeutic intervention in the absence of EMR during the TKI treatments is described in the ELN 2013 recommendations.^1^

TFR (treatment-free remission) is the discontinuation of TKI in the superior-TKI responder patient with CML. The deeper molecular responses (MR4, MR4.5, MR5) detected during at least two years of monitoring in CML are candidates for TFR. MR4 can be achieved by a BCR-ABL expression < 0.01%, MR4.5 by <0.0032% BCR-ABL^IS^, and MR5 by <0.001% BCR-ABL^IS^. Young and low prognostic risk CML patients are candidates of first line 2^nd^ generation TKIs with the aim of drug discontinuation in their future life.^16^

Mutational analyses shall only be performed in patients with suboptimal responses, warnings, and failures in CML cases subject to the alterations in the treatment strategies.^1^ Mutations detected during the TKI therapy may be resulted in drug switches based on the nature of the mutation. T315I, Y253K, E255K, E255V, F359V, F359C, are the mutations poorly sensitive to nilotinib; whereas T315I, T315A, F317L, F317C, V299L are the mutations poorly sensitive to dasatinib. T315I is a unique mutation making the CML patient irresponsive to all available TKIs but ponatinib and allografting.^22^–^29^

Patients with advanced phase (AP/BC) CML are currently treated with the most powerful TKI^30^ available (dasatinib^31^ or ponatinib^32^) and multi-agent chemotherapy before allografting. Monitoring of those patients is also problematic. Durable hematological, cytogenetic, molecular responses are hard to be obtained in the CML patients with advanced phase (AP/BC) disease. Although durable hematologic, cytogenetic and molecular responses can be hardly obtained in AP and particularly in BP patients, the definition of the responses should be the same as for CP patients. Proper therapeutic interventions in advanced phase CML are described elsewhere.^1^,^2^

## Practical Problems in the Long-Term Monitoring of TKI Treatment in CML

CHR, early CCyR, faster MMR, and the deeper, durable molecular responses (MR4, MR4.5, MR5) are the ultimate goals of the TKI-receiving patients with CML. Critical evaluations of the CML patients to hit those targets shall be made at the baseline, and at the 3^rd^ month, 6^th^ month, 12^th^ month, and thereafter the TKI administration. There are some practical and technique-related problems during the hematological, cytogenetic, molecular monitoring of TKI treatment in CML. Clinical significances of those incidences during the long-term monitoring in CML are indicated below;

Hydroxyurea treatment, especially in sustained high doses, before the initiation of TKI regimen, could obscure the evaluation of CHR and baseline CML disease risk profile of the patient. Before the TKI decision, the baseline assessments of the de novo CML patient shall include exact medical diagnosis of CML, basic laboratory evaluation covering CBC and peripheral blood smear (PBS), bone marrow cytology, conventional cytogenetics and/or FISH analyses for Ph+ chromosome, and qualitative molecular analyses for the BCR-ABL.^1^ Tumor load and disease phase should be defined. Newly diagnosed CP-CML patients should be stratified based on the Sokal, Euro/Hasford and EUTOS CML prognostic scoring systems.^33^ Hydroxyurea can affect CBC, PBS, spleen size, bone marrow cellularity, the quality of metaphases, and essential parameters of the Sokal, Euro/Hasford and EUTOS CML prognostic scoring systems. Therefore, baseline CML disease risk profile of the patient shall be obtained before the hydroxyurea and/or TKI were administered to the patient.

**Figure 1:**
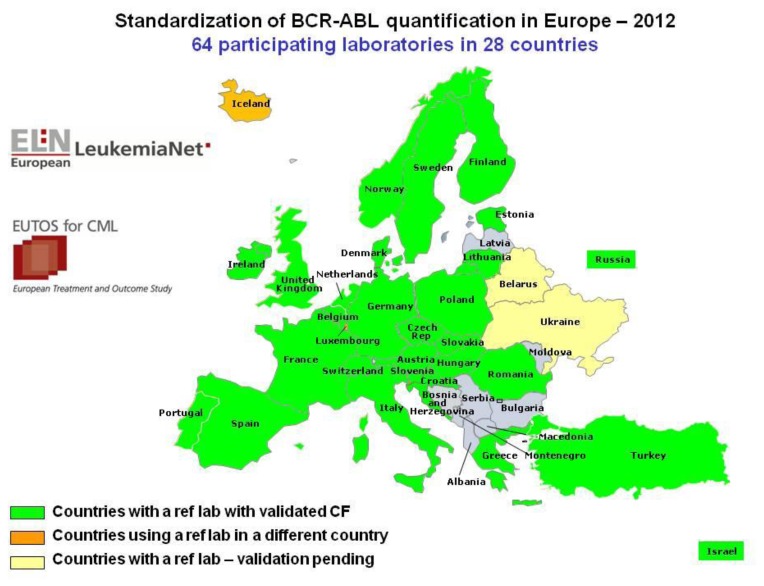
Standardization of BCR-ABL quantification in Europe have been performed by European LeukemiaNet (ELN) and EUTOS. The 2012 status of the BCR-ABL standardization within 64 participating laboratories in 28 countries including the Mediterranean land is depicted.

The estimated ratio of BCR-ABL/ABL is highly technique-dependant. Many laboratories in the world are not yet qualified for the international harmonization of scale (IS). Standardization of BCR-ABL quantification in Europe have been performed by European LeukemiaNet (ELN) and the European Treatment, and Outcome Study (EUTOS).^34^ The high efficacy of the TKI treatment of CML has prompted the need for accurate methods to monitor response at levels below the landmark of CCyR. Quantification of BCR-ABL transcripts has proven to be the most sensitive method available and has shown prognostic impact with regard to progression-free survival. The variations in the methods used to quantify BCR-ABL made it difficult to compare results between laboratories. ELN program harmonized the reporting of results according to the IS in Europe. The ELN recommendations for the propagation of the IS by national or regional laboratory networks.^34^ The 2012 status of the BCR-ABL standardization within 64 participating laboratories in 28 countries including the Mediterranean land is depicted in Figure 1.

Regarding the EMR, the challenges for the widespread routine use of the 10% BCR-ABL transcript cut-off at the 3^rd^ month of TKI are present. High ratio values on IS scale, housekeeping control gene problem, variations in the samples, delays in the exact molecular assessment time after TKI and early unexpected variation kinetics of response in individual CML patients complicate the interpretation of the 10% BCR-ABL transcript cut-off at the 3^rd^ month of TKI. Likewise, the tumor burden at diagnosis, prognostic scoring, gene profile, cytoreduction before TKI, treatment adherence, and numerous confounding effects may obscure the real-life decision at the 3^rd^ month of TKI outside the clinical trials. Nevertheless, obtaining faster, deeper and durable molecular responses particularly MMR are essential for the patient with CML in the TKI era. Proper therapeutic interventions based on the molecular responses are described in the ELN 2013 recommendations.^1^

The cytogenetic analyses also have technique-dependant problems. Obtaining the CBA of the bone marrow cell metaphases at 3 and 6 months, then every 6 months until the CCyR^1^ could not be possible in all cases of CML under TKI. Invasive nature of the bone marrow aspiration/biopsy could represent another clinical problem. FISH of the blood cells can substitute CBA if bone marrow cells cannot be obtained for the definition of CCyR. The standardization about the sensitivity level of FISH has improved. Nevertheless, obtaining earlier and stable cytogenetic responses particularly CCyR are essential for the patient with CML in the TKI era. Proper therapeutic interventions based on the cytogenetic responses are described in the ELN 2013 recommendations.^1^

CML treatment may be modelled on the individual disease and patients characteristics (risk, molecular profile, age, co-morbidities, aggressive clinical course, etc.). Therefore, the CML monitoring strategy to detect the response to TKI may also be varied and tailored on an individual basis. Drug tolerability, patient compliance of TKI, physician adherence to TKI, and off-target TKI complications should always be monitored during the CML treatment. Otherwise, late, off-target complications of TKI (lung toxicity,^35^ cardiac toxicity,^36^,^37^ metabolic syndrome^21^, bone toxicity,^38^ arterial and venous occlusive events,^39^ pancreas toxicity^1^, and others) may limit the benefits of the given TKI. Proper therapeutic interventions based on the therapeutic monitoring of the CML patients and TKI drugs are described in the ELN 2013 recommendations.^1^

## Future Perspectives in the Monitoring of TKI Treatment in CML

Novel recent investigations for the de novo CML patients have searched the validity of gene expression profiling, genetic polymorphisms, next generation genomics, multi-drug resistance genes (MDR, OCT1),^15^ fusion transcripts and pre-existing BCR-ABL kinase domain mutations. The cessation of the TKI therapy with the aim of cure,^40^ stem cell depletion, stem cell exhaustion, immunological control of the disease will be the future therapeutic tools of CML. The improvements in the international harmonization of scale about the molecular monitoring would be very important in the TFR stage of CML with the intention to cure the disease.

## Figures and Tables

**Table 1:** The definition of the hematologic, cytogenetic and molecular responses in CML.

Type of Response		Definition
CHR	Complete Hematologic Response	Normal differential, WBC & platelets ≤ ULN
MCyR	Major cytogenetic Response	0–35% Ph+marrow metaphases
CCyR	Complete Cytogenetic Response	0% Ph+marrow metaphases
MMR	Major Molecular Response	BCR-ABL/ABL ≤ 0.1% (International Scale)
MR^4.0^		BCR-ABL/ABL ≤ 0.001% (IS) “4-log reduction”
MR^4.5^		BCR-ABL/ABL ≤ 0.003% (IS) “4.5-log reduction”
CMR	Complete Molecular Response	Undetectable BCR-ABL (test of sensitivity ≥4.5 logs)

**Table 2:** The ideal responses to the tyrosine kinase inhibitor (TKI) treatment detectable during the long-term monitoring of chronic myeloid leukemia (CML).

	Ideal response at the 3rd month of TKI	Ideal response at the 6th month of TKI	Ideal response at the 12th month of TKI	Ideal response after one year of TKI and thereafter
**Hematological monitoring**	CHR	CHR	CHR	CHR
**Cytogenetic monitoring**	MCyR	CCyR	CCyR	CCyR
**Molecular monitoring**	BCR-ABL/ABL below 10%	BCR-ABL/ABL below 1%	MMR	better than MMR; MR4, MR4.5, MR5

CHR; complete hematological response. MCyR; major cytogenetic response. CCyR; complete cytogenetic response. MMR; major molecular response.
